# Carbon-Nitride-Based Materials for Advanced Lithium–Sulfur Batteries

**DOI:** 10.1007/s40820-022-00954-x

**Published:** 2022-11-14

**Authors:** Wenhao Sun, Zihao Song, Zhenxing Feng, Yaqin Huang, Zhichuan J. Xu, Yi-Chun Lu, Qingli Zou

**Affiliations:** 1 State Key Laboratory of Chemical Resource Engineering, Beijing University of Chemical Technology, Beijing, 100029 People’s Republic of China; 2 Electrochemical Energy and Interfaces Laboratory, Department of Mechanical and Automation Engineering, The Chinese University of Hong Kong, Hong Kong Special Administrative Region, 999077 People’s Republic of China; 3 School of Materials Science and Engineering, Nanyang Technological University, Singapore, 639798 Republic of Singapore; 4 School of Chemical, Biological, and Environmental Engineering, Oregon State University, Corvallis, OR 97331 USA

**Keywords:** Lithium–sulfur batteries, Carbon nitride, Polysulfide conversion, Shuttle effect, Anode protection

## Abstract

Lithium–sulfur (Li–S) batteries are promising candidates for next-generation energy storage systems owing to their high energy density and low cost. However, critical challenges including severe shuttling of lithium polysulfides (LiPSs) and sluggish redox kinetics limit the practical application of Li–S batteries. Carbon nitrides (C_*x*_N_*y*_), represented by graphitic carbon nitride (g-C_3_N_4_), provide new opportunities for overcoming these challenges. With a graphene-like structure and high pyridinic-N content, g-C_3_N_4_ can effectively immobilize LiPSs and enhance the redox kinetics of S species. In addition, its structure and properties including electronic conductivity and catalytic activity can be regulated by simple methods that facilitate its application in Li–S batteries. Here, the recent progress of applying C_x_N_y_-based materials including the optimized g-C_3_N_4_, g-C_3_N_4_-based composites, and other novel C_*x*_N_*y*_ materials is systematically reviewed in Li–S batteries, with a focus on the structure–activity relationship. The limitations of existing C_*x*_N_*y*_-based materials are identified, and the perspectives on the rational design of advanced C_*x*_N_*y*_-based materials are provided for high-performance Li–S batteries.

## Highlights

- The recent advances in C_*x*_N_*y*_-based materials including the optimized g-C_3_N_4_, g-C_3_N_4_-based composites, and other novel C_*x*_N_*y*_ materials are summarized.
- The applications of C_*x*_N_*y*_-based materials in Li–S batteries are systematically discussed with a focus on the structure–activity relationship.
- The perspectives on the rational design of advanced C_*x*_N_*y*_-based materials for high-performance Li–S batteries are provided.

## Graphical abstract

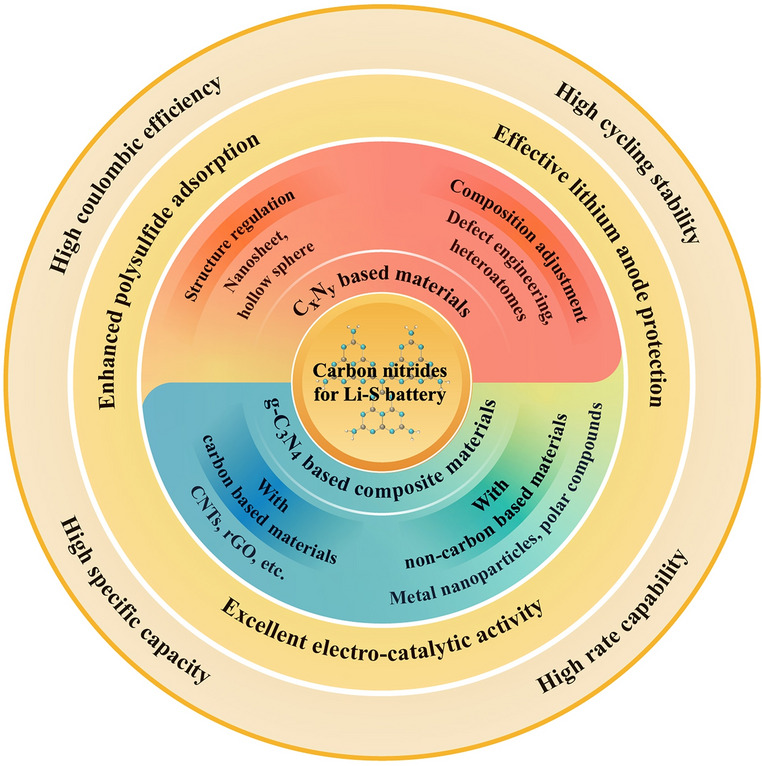

## Introduction

The continuously increasing demands for sustainable energy and severe environmental crisis have boosted the development of various advanced energy technologies around the world, with the purpose of efficient utilization and storage of renewable energy [1, 2]. High energy density and economical rechargeable batteries are the key components of these advanced energy technologies [3–5]. Operated based on lithium ion (Li-ion) intercalation chemistry, Li-ion batteries have enjoyed great success in powering commercial portable electronics and electric vehicles [6]. However, the limited capacity of electrode materials and their high cost hinder the penetration of traditional Li-ion batteries in large-scale emerging fields. Therefore, it is increasingly important to develop electrochemical energy storage devices with higher energy density and lower cost [7–9].

**Fig. 1:**
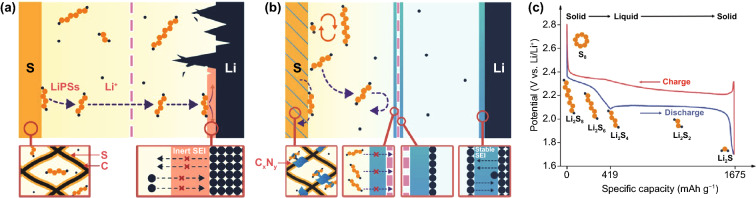
Schematic illustration of **a** the challenging issues in common Li–S batteries and **b** the improved performance for modified Li–S batteries with the introduced C_x_N_y_ additive in different components. **c** Typical discharge/charge voltage profiles of Li–S batteries

Lithium–sulfur (Li–S) batteries are considered one of the most promising energy storage systems beyond Li-ion batteries due to their high energy density and low cost [10]. Typically, Li–S batteries consist of elemental sulfur (S_8_) cathodes and Li anodes, as shown in Fig. 1a. Based on the multi-electron conversion mechanism between S_8_ and Li metals (S_8_ + 16Li ↔ 8Li_2_S) [11, 12], Li–S batteries deliver high theoretical specific capacity of 1675 mAh g^−1^ and specific energy of 2,600 Wh kg^−1^, which is 2–5 times that of Li-ion batteries [13]. The widely accepted reaction mechanism of Li–S batteries is shown in Fig. 1c. During the discharge process, solid S_8_ is firstly reduced to soluble lithium polysulfides (LiPSs, usually denoted as Li_2_S_n_, 2 < n ≤ 8) in a first discharge plateau at around 2.35 V and then continues to be reduced to solid lithium sulfide (Li_2_S) in a second discharge plateau at around 2.1 V. Due to the involved solid–solid conversion between Li_2_S_2_ and Li_2_S, the corresponding reaction kinetics performs sluggish. During the subsequent charge process, Li_2_S is reconverted to LiPSs and finally to S_8_, forming a reversible cycle [14].

Due to inherent properties of the Li–S reaction, the commercial applications of Li–S batteries are limited by three main obstacles (Fig. 1a). (i) The insulating and insoluble nature of S_8_ and Li_2_S limits their utilization efficiency and redox kinetics [15–17], especially for the deposition of solid Li_2_S, which is considered to be the rate-limiting step of the whole discharge process due to the sluggish kinetics in the solid–solid conversion from Li_2_S_2_ to Li_2_S, resulting in low capacity and low rate performance of Li–S batteries; (ii) long-chain LiPSs intermediates are soluble in organic electrolyte, which leads to their shuttling to the Li anode and thus low coulombic efficiency, high self-discharge, and passivation of the Li anode surface with the continuous reaction with LiPSs [13, 18]; (iii) due to the density difference between S_8_ and Li_2_S, the cathode encounters a large volume change (≈80%) during the discharge and charge process [19, 20], leading to irreversible damage of the electrode structure and rapid capacity fading; (iv) due to the uneven Li deposition, the generated Li dendrites may pierce the separator, resulting in internal short circuiting and even explosion [13].

S composite cathodes [21–24] and functional interlayers/separators [25–28] based on advanced materials could mitigate these obstacles and achieve high-performance Li–S batteries. Adsorption by a large surface area and abundant active sites could effectively immobilize LiPSs, and high catalytic activity could enhance the reaction kinetics [29–31]. Moreover, highly conductive, interconnected, and flexible structure could promote the utilization of the insulating active materials (S_8_ and Li_2_S) and mitigate the impact of volume expansion [32–34]. Carbon nitride (C_x_N_y_) is a kind of ordered semiconductor material with strong absorption capability, high catalytic activity, excellent stability, low cost, and environment friendliness, rendering it a promising additive for Li–S batteries [35, 36]. As shown in Fig. 1b, represented by graphitic carbon nitride (g-C_3_N_4_), its high N content provides abundant active sites for LiPSs’ immobilization. Moreover, the heptazine units of g-C_3_N_4_ contain high levels of pyridinic N, which can provide lone pair electrons to promote electrochemical reactions [37, 38]. Besides, the high N content of g-C_3_N_4_ endows it with good capability to homogenize Li ion deposition with the strong affinity between Li and N atoms, and the stable g-C_3_N_4_ coating on Li anode can physically inhibit the growth of Li dendrites and guarantee the fast transport for Li ions [39, 40]. Although the original g-C_3_N_4_ bulk shows limited electronic conductivity and surface area [34, 41, 42], these properties can be improved by regulating its structure and properties with simple methods. With various precursors and synthesis conditions, g-C_3_N_4_ could be synthesized with different C/N ratios, surface area, porosity, nanostructure shapes, and morphologies [43–46].

In this review, we present recent advances in C_x_N_y_-based materials applied in Li–S batteries, including the optimized g-C_3_N_4_, g-C_3_N_4_-based composite materials, and other novel C_x_N_y_ materials. We systematically summarized their synthetic methods, structures, properties, and effects on Li–S batteries, with a focus on the structure–activity relationship. Based on an extensive analysis of literature, we identified the limitations of existing C_x_N_y_-based materials and provided our perspective on the rational design of advanced C_x_N_y_-based materials for high-performance Li–S batteries.

## Basics of Representative Carbon Nitride: g-C_3_N_4_

C_x_N_y_ material, represented by g-C_3_N_4_, was firstly reported in the nineteenth century [37]. Due to its unique structure and properties, g-C_3_N_4_ has been widely applied in various fields ever since, such as photocatalysis [47], carbon dioxide capture [48, 49], and energy storage, for example, Li–S batteries [35].

### Structures and Properties

**Fig. 2:**
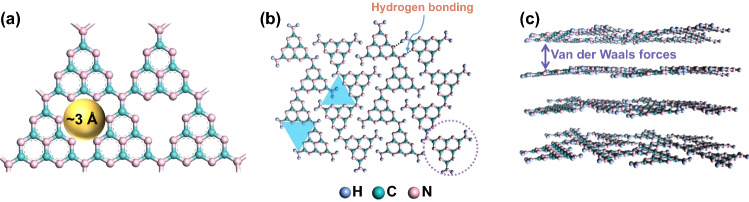
**a** Tri-s-triazine unit of g-C_3_N_4_. **b** Plane repeats and **c** π–π stacking structure of g-C_3_N_4_ [37]. Copyright 2021, Wiley–VCH

Common g-C_3_N_4_ exhibits a graphene-like nanosheet structure. In the lamellas, tri-s-triazine rings as basic units [37] are composed of *sp*^2^ hybrid conjugated C and N atoms and further connected by hydrogen bonds between NH and/or NH_2_ groups on ring edges [50, 51], as shown in Fig. 2a-b. The connection of multiple basic units constructs angstrom pore structure in the lamellas with a diameter of around 3 Å, which is larger than that of Li^+^ and smaller than that of soluble LiPSs [52], leading to the restricted "shuttle effect". Between these lamellas, there exists a weak van der Waals force (Fig. 2c) [53], which provides a stronger interlamellar binding ability and smaller stacking distance (0.319 nm) compared to that of graphite (0.335 nm) [54].

g-C_3_N_4_ has high stability and flexibility, which attribute to its intra- and inter-lamella structure. The aromatic heterocyclic ring in the lamella of g-C_3_N_4_ ensures its high thermal stability. g-C_3_N_4_ can withstand a high temperature of around 600 °C in the air without obvious degradation observed [51]. The strong inter-lamellar binding ensures its high chemical stability. g-C_3_N_4_ is insoluble in most acid/alkali water solutions or organic solutions [55, 56]. Moreover, g-C_3_N_4_ shows high flexibility, which is conducive to alleviating the volume change of electrodes during the charge and discharge process [57].

**Fig. 3:**
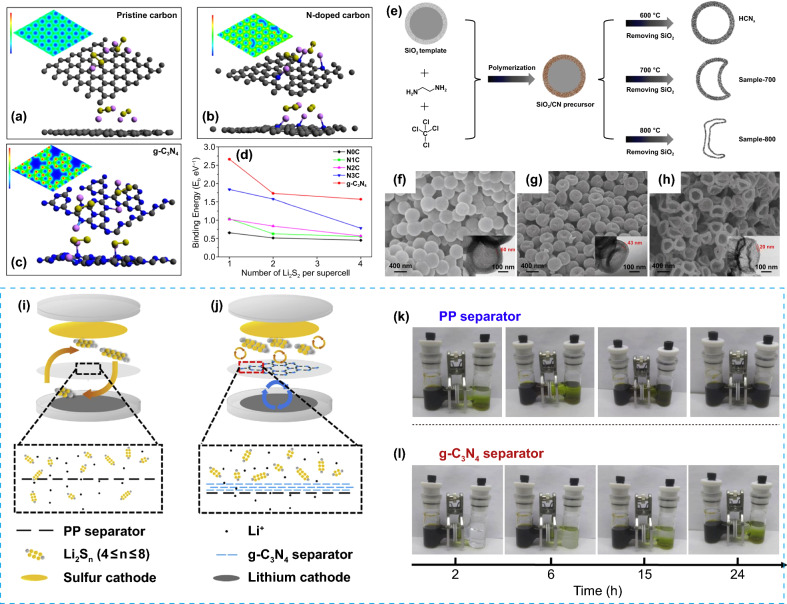
The most stable adsorption models of two Li_2_S_2_ molecules on **a** pristine carbon, **b** N-doped carbon, and **c** g-C_3_N_4_; the insets in the top left show the two-dimensional deformation charge distributions of the corresponding matrixes without Li_2_S_2_ (red for receiving electron and blue for giving electron). **d** The binding energies (per Li_2_S_2_) of Li_2_S_2_ molecules on different matrixes as the number of Li_2_S_2_ changes [35]. Copyright 2016, American Chemical Society. **e** Schematic illustration of the preparation process of HCN_x_, sample-700 and sample-800. **f** SEM and TEM images of HCN_x_, **g** sample-700 and **h** sample-800 [45]. Copyright 2017, Wiley–VCH. Schematic illustration of Li–S cells with **i** PP separator and **j** g-C_3_N_4_ separator. H-shaped LiPSs permeation devices with **k** PP separator and **l** g-C_3_N_4_ separator [64]. Copyright 2018, Elsevier

More importantly, g-C_3_N_4_ performs strong LiPSs adsorption capability due to its high N content (60.87 wt%, in theory) and various types of N-site, including bridged N, graphitic N, and pyridinic N. The strong interaction between N and Li [35, 36] effectively immobilizes LiPSs and further accelerates the redox reaction of S species, improving the rate capability of Li–S batteries [58]. The adsorption performances of pristine carbon, N-doped carbon, and g-C_3_N_4_ to LiPSs were compared by the first-principles calculation [35]. Figure 3a-c displays the 2D deformation charge distribution of various substrates (red for receiving electron and blue for giving electron) and the most stable adsorption configurations of Li_2_S_2_ molecules on their surfaces. The pristine carbon shows evenly distributed positive charges on each C site and adsorbs Li_2_S_2_ without special binding bonds, while both N-doped carbon and g-C_3_N_4_ perform negative charges on the N sites and adsorb Li_2_S_2_ by forming a Li-N bond with the average distance between g-C_3_N_4_ and Li_2_S_2_ as short as 2.06 Å. In addition, as shown in Fig. 3d, g-C_3_N_4_ shows higher binding energy for Li_2_S_2_, confirming that g-C_3_N_4_ can provide rich active sites (pyridinic N) for the adsorption of LiPSs with high intrinsic polarity.

Besides, the high N content of g-C_3_N_4_ endows it with high affinity with Li ions, which can ensure the uniform deposition of Li ions [40]. Moreover, the shear modulus of the g-C_3_N_4_ layer is about 21.6 Gpa, which is higher than that of Li metal (~ 4.9 Gpa), thus physically inhibiting the growth of Li dendrites [39].

### Synthesis Methods and Applications in Li–S Batteries

The synthesis methods of g-C_3_N_4_ reported mainly include the direct condensation method and template method. By heating the nitrogen-containing precursor such as melamine at a certain temperature, direct condensation is a simple and commonly applied method to fabricate 2D g-C_3_N_4_ nanosheet. With this method, the properties of g-C_3_N_4_ such as specific surface area could be easily regulated by changing the types of precursor and heating temperature. Different precursors lead to different reaction processes and different by-products, which affect the structure of g-C_3_N_4_. For example, the evolution of NH_3_ gas during the calcination of melamine (g-C_3_N_4_ precursor) could lead to a g-C_3_N_4_ product with a porous structure [59]. Heating temperature mainly affects the reaction rate. An excessive temperature would lead to the collapse of g-C_3_N_4_ structure. Li et al. prepared g-C_3_N_4_ using cyanuric acid (TTCA) as the precursor [60]. The g-C_3_N_4_ product shows a porous structure with high specific surface area and high N doping content (up to 56.87 wt%), which facilitate the fast ion transfer and LiPSs immobilization. Furthermore, Yao et al. systematically studied the effect of precursors [43] and pyrolysis temperature [61] on the properties of g-C_3_N_4_. Among the g-C_3_N_4_ materials prepared with different precursors, including urea, melamine, thiourea, and dicyandiamide, urea-based g-C_3_N_4_ shows the highest specific surface area (~ 93 m^2^ g^−1^). For the pyrolysis temperature, g-C_3_N_4_ synthesized at 550 °C shows the highest specific surface area with a rich mesoporous lamellar structure. These results were later reconfirmed by Versaci et al. [44]. In addition, they further proved that g-C_3_N_4_ prepared at 550 °C with urea had a high content of -NH_2_ group, which was conducive to the immobilization of soluble LiPSs.

Based on the direct condensation mechanism, the template method is introduced to fabricate g-C_3_N_4_ with 3D secondary structures such as hollow or core–shell structures, which could not only provide high surface area but also accommodate the volume change during cycling. The template and reaction temperature selected are important, which affect the structure and properties of the product. Silica is a common template applied. A hollow g-C_3_N_4_ material was prepared using mesoporous silica as a template and further constructed into an S@C_3_N_4_ composite cathode with a core–shell structure [62]. In addition, Han et al. used silica microspheres as a hard template and investigated the effect of calcination temperature on the structure of the synthesized hollow g-C_3_N_4_ microspheres [45]. With calcination temperatures set as 600, 700, and 800 °C, the synthesis procedure is shown in Fig. 3e. According to the SEM images of as-prepared g-C_3_N_4_ (Fig. 3f-h), the thickness of the shell decreases, and the microsphere structure collapses as the temperature increases from 600 to 800 °C. This could be related to the excessively decompose of the precursors at high temperatures. As a result, the cell with g-C_3_N_4_ prepared in 600 °C as the S host exhibited a low capacity fading rate of 0.076% per cycle after 500 cycles at 0.5C.

Generally, g-C_3_N_4_ is applied as an additive to the S cathode. Li et al. fabricated composite cathodes with g-C_3_N_4_ and S, which exhibited a high capacity of 1200 mAh g^−1^ at 0.2C and maintained a high capacity of 800 mAh g^−1^ after 100 cycles with the coulombic efficiency above 99.5% [60]. Yet some studies also use g-C_3_N_4_ to construct multifunctional layers on the cathode or separator to limit the diffusion of LiPSs. Li et al. coated a layer of g-C_3_N_4_ nanosheets on the surface of the S cathode (S-C_3_N_4_) by the spraying method [63]. This unique design has the following advantages: (1) the g-C_3_N_4_ layer has a strong chemical adsorption capability for LiPSs, which can limit LiPSs shuttling and alleviate the self-discharge phenomenon; (2) spraying technology ensures the uniformity of the coating, and it is easy to large-scale production with the controlled thickness. Therefore, the cell with an S-C_3_N_4_ composite cathode displayed a high capacity of 630 mAh g^−1^ at 5C. Similarly, Xie et al. coated ultra-thin g-C_3_N_4_ nanosheets on the commercial polypropylene (PP) separator (g-C_3_N_4_ separator) by using the vacuum filtration technology [64], which effectively prevents LiPSs from diffusing across the separator but allows lithium ions to pass freely (Fig. 3i-j). Moreover, the LiPSs permeation test also showed the strong restriction effect of g-C_3_N_4_ for LiPSs diffusion (Fig. 3k-l). Thus, the cell with a g-C_3_N_4_ separator performed a high capacity of 829 mAh g^−1^ after 200 cycles at 0.2C.

## Optimization of g-C_3_N_4_

With various synthesis methods, g-C_3_N_4_ could perform different microstructures with enhanced specific surface area. Beyond this, the LiPSs absorption capability, catalytic activity, and electron conductivity of g-C_3_N_4_ could be further improved via defect engineering and heteroatom doping. Defect engineering plays an important role in adjusting the atomic distribution and surface properties of nanomaterials and has widespread application in various fields including hydrogen evolution reaction [65], oxygen evolution reaction [66], and carbon dioxide reduction reaction [67]. Heteroatom doping is also an effective method to regulate the polarity of carbon materials, and various heteroatoms including nonmetal atoms and metal-single atoms have been studied extensively [68–70]. In particular, the introduction of metal single atoms with unsaturated coordination environments, unique electronic structures, and high surface free energy could significantly enhance the catalytic activity of the materials [71–73]. In recent years, defect engineering and heteroatom doping have attracted more and more attention in Li–S systems due to their significant potential in inhibiting LiPSs shuttling and promoting the redox chemistry [74–77].

### Defect Engineering

**Fig. 4:**
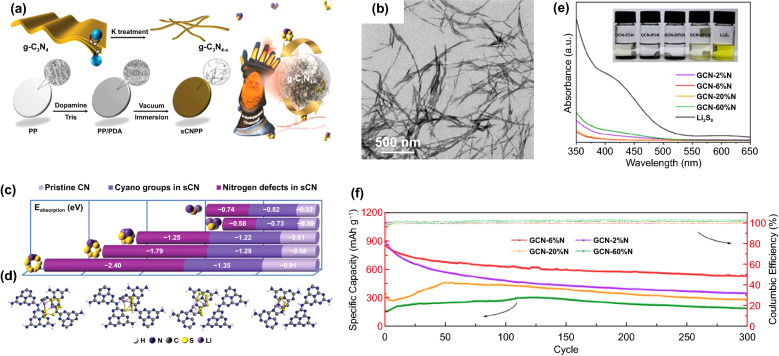
**a** Schematic illustration of the preparation process of sCN/PDA/PP separator. **b** TEM images of sCN. **c** Adsorption energies for Li_2_S, Li_2_S_2_, Li_2_S_4_, Li_2_S_6_ and Li_2_S_8_ on pristine CN, the cyano group of sCN and the N vacancy of sCN. **d** The stable adsorption models of Li_2_S_8_ or Li_2_S_4_ on the cyano group of sCN and the N vacancy of sCN [58]. Copyright 2021, Wiley–VCH. **e** UV–Vis spectra of a bare Li_2_S_8_ solution and Li_2_S_8_ solutions with different GCN materials after aging for 24 h; the inset is the corresponding optical photograph. **f** Cycling performance of cells with different GCN/S cathodes at 0.5C [46]. Copyright 2020, American Chemical Society

With a certain proportion of N defects, g-C_3_N_4_ materials show enhanced adsorption and catalytic performance of LiPSs. Huang et al. prepared ultrafine spindle g-C_3_N_4_ (sCN) with N defects by K treatment (Fig. 4a) [58]. Compared with the original g-C_3_N_4_, the sCN performs spindle-like morphology (Fig. 4b) and an obvious different molecular structure with a large number of defects manifested as N vacancies or cyano groups (Fig. 4c-d). The introduction of N defects increases the polarity of sCN, which leads to 2–3 times increased LiPSs binding energy compared with that of the original g-C_3_N_4_. Therefore, the Li–S cell with sCN modified separator delivered a high initial capacity of 637 mAh g^−1^ at 5C and a low capacity fading rate of 0.05% per cycle after 500 cycles. Besides, various g-C_3_N_4_ materials with different defect structures, concentrations, and preparation methods have been reported, which obviously improve the performance of Li–S batteries [78, 79]. However, excessive N defects could destroy the structure of g-C_3_N_4_ and thus decrease its electron transport and LiPSs adsorption capability. According to Du et al. [46] (Fig. 4e), as the N content decreases from the original 60% (GCN-60%N) to 6% (GCN-6%N), the content of defect increases, which leads to an increased LiPSs adsorption capability of the material. It is worth noting that when the nitrogen content continues to drop below 6%, the adsorption capability of the material (GCN-2%N) for LiPSs begins to decrease. This could be related to the destruction of the material structure, which is also reflected in the performance of Li–S batteries. The cell with optimized GCN-6%N/S composite cathode displayed a high initial capacity of 852.2 mAh g^−1^ at 0.5C and retained a reversible capacity of 532.4 mAh g^−1^ after 300 cycles (Fig. 4f).

### Heteroatoms Doping

Heteroatom-doped g-C_3_N_4_, including nonmetal atom- and metal single-atom-doped materials, are reported to be applied in Li–S batteries with enhanced cycling performance and distinct working mechanisms.

**Fig. 5:**
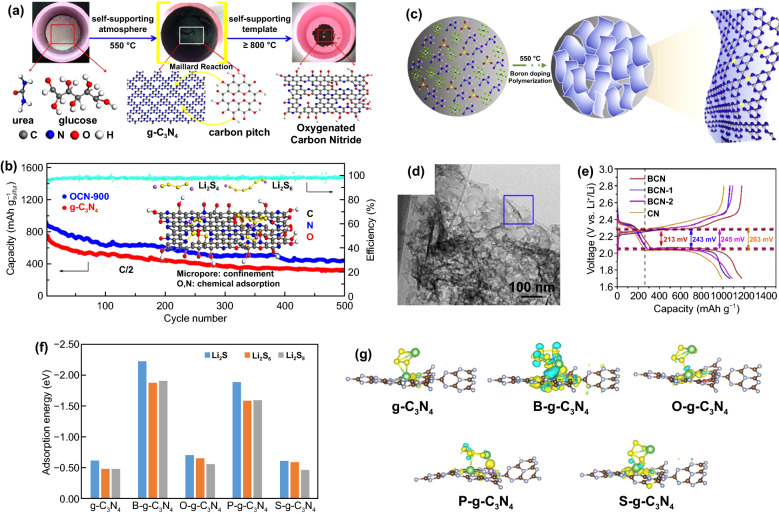
**a** Schematic illustration of the preparation process and structure of OCN. **b** Cycling performance of cells with g-C_3_N_4_ and OCN-900 as sulfur hosts at 0.5 C [80]. Copyright 2015, American Chemical Society. **c** Schematic illustration of the preparation process and structure of BCN. **d** STEM image of BCN. **e** Charge–discharge voltage profiles of cells with different modified separators [81]. Copyright 2020, Elsevier. **f** Adsorption energies for Li_2_S_4_, Li_2_S_6_ and Li_2_S_8_ on g-C_3_N_4_, B-g-C_3_N_4_, O-g-C_3_N_4_, P-g-C_3_N_4_ and S-g-C_3_N_4_. **g** Differential charge densities of Li_2_S_4_ adsorbed on g-C_3_N_4_, B-g-C_3_N_4_, O-g-C_3_N_4_, P-g-C_3_N_4_ and S-g-C_3_N_4_ [82]. Copyright 2021, Elsevier

Nonmetal atom doping, such as N, S, O, P, and B, enhances the electronic conductivity and the LiPSs absorption capability of g-C_3_N_4_. Liu et al. [80] prepared O-doped g-C_3_N_4_ nanosheets (OCN) by one-step self-supported solid-state pyrolysis (OSSP) technique with urea as the precursor and glucose as the oxygen source (Fig. 5a). The introduction of O atoms into g-C_3_N_4_ promotes the chemical interactions with LiPSs by forming Li–O bonds. Thus, the cell with OCN/S composite cathode performed a high capacity of 447.3 mAh g^−1^ after 500 cycles at 0.5C with the capacity fading rate of 0.1% per cycle (Fig. 5b). Zhang et al. prepared P-doped g-C_3_N_4_ (PCN), which was used as the S host to enhance the performance of Li–S batteries [83]. According to density functional theory (DFT) calculation results [83, 84], both OCN and PCN have higher conductivity and stronger adsorption capability for LiPSs compared with original g-C_3_N_4_, which is conducive to improving the S utilization efficiency. In addition to O and P, B-doped g-C_3_N_4_ nanosheets (BCN) were prepared by a one-pot thermal condensation method and used as functional separator coating for Li–S batteries [81]. As shown in Fig. 5c, in the heat treatment process, the g-C_3_N_4_ bulk was exfoliated to g-C_3_N_4_ nanosheets due to the blowing erosion caused by the decomposition of ammonium chloride. At the same time, B atoms were successfully doped into g-C_3_N_4_ matrix with N-B-N bonds. The TEM images in Fig. 5d show that BCN performs the wrinkled and irregular lamellar structure. The low polarization overpotential and high capacity of Li–S cells with BCN-coated separator at 0.5C (Fig. 5e) suggest the improvement of S utilization efficiency and redox kinetic.

Yanmsang et al. investigated the adsorption capabilities and mechanisms of g-C_3_N_4_-doped with different heteroatoms (B, O, P and S) for LiPSs on the molecular level by DFT calculations [82]. As shown in Fig. 5f-g, the B-doped g-C_3_N_4_ (B-g-C_3_N_4_) shows the strongest adsorption capability for LiPSs among investigated g-C_3_N_4_ materials. The result can be attributed to the lower capability of B atoms to attract electrons than C atoms, resulting in more negative charge accumulation around pyridinic N atoms and thus facilitating charge transfer between g-C_3_N_4_ and LiPSs.

Metal single-atom doping, such as Fe, Co, and Ni, also improves the adsorption and electrical conductivity of g-C_3_N_4_ [85–87]. In addition, the metal atoms as electropositive active sites could directly interact with LiPSs, which largely improves the catalytic activity of doped g-C_3_N_4_ for LiPSs redox reactions [86].

**Fig. 6:**
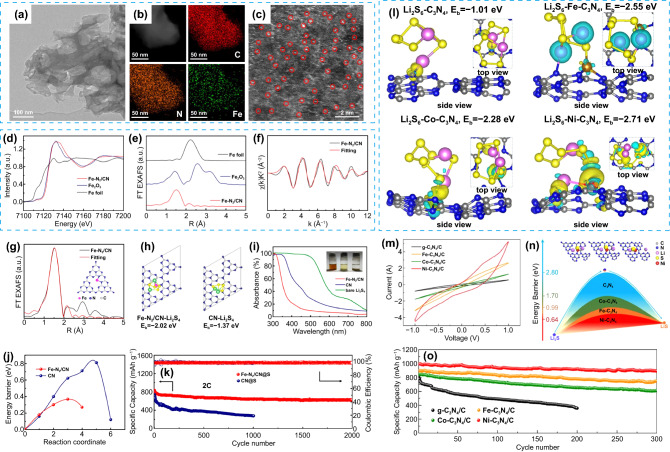
**a** TEM image and **b** corresponding EDS mappings and **c** HAADF-STEM image of Fe-N_2_/CN. **d** Fe K-edge XANES spectra and **e** Fourier transformation of Fe K-edge EXAFS spectra of Fe-N_2_/CN, Fe foil, and Fe_2_O_3_. **f** Fe k-space EXAFS curve and corresponding fitting curve, and **g** Fe r-space EXAFS curve and corresponding fitting curve of Fe-N_2_/CN. **h** Optimized structures and binding energies of Li_2_S_4_ adsorbed on Fe-N_2_/CN and CN surfaces. **i** UV–vis spectra of Li_2_S_4_ solution with CN and Fe-N_2_/CN. **j** Decomposition energy barriers of Li_2_S on Fe-N_2_/CN and CN surfaces. **k** Cycling performance of cells with CN@S and Fe-N_2_/CN@S cathodes at 2C [85]. Copyright 2020, American Chemical Society. **l** Differential charge densities of Li_2_S_6_ adsorbed on C_3_N_4_, Fe-C_3_N_4_, Co-C_3_N_4_ and Ni-C_3_N_4_. **m** CV curves of symmetric cells with g-C_3_N_4_/C, Fe-C_3_N_4_/C, Co-C_3_N_4_/C and Ni-C_3_N_4_/C modified separators. **n** Decomposition barriers of Li_2_S on C_3_N_4_, Fe-C_3_N_4_, Co-C_3_N_4_ and Ni-C_3_N_4_. **o** Cycling performance of cells with g-C_3_N_4_/C, Fe-C_3_N_4_/C, Co-C_3_N_4_/C and Ni-C_3_N_4_/C modified separators at 0.5 A g^−1^ [86]. Copyright 2019, Elsevier

Fe atom-doped g-C_3_N_4_ (Fe-N_2_/CN) material with a hierarchical porous lamellar structure was successfully prepared by Qiu et al. (Fig. 6a-b) [85]. The uniform pyridinic N sites of g-C_3_N_4_ control the coordination structure of Fe-NC. As shown in Fig. 6c, a large number of independent Fe atoms with the size of about 2 Å are evenly distributed in obtained g-C_3_N_4_. According to the X-ray absorption near-side structure spectra (XANES) and Fourier transform of Fe K-edge extended X-ray absorption fine structure (EXAFS) spectra of Fe-N_2_/CN (Fig. 6d-g), Fe atoms on g-C_3_N_4_ are positively charged and coordinated with two N atoms through N–Fe–N bond. These Fe-N_2_ unsaturated sites show not only stronger LiPSs adsorption capability with higher binding energy (Fig. 6h-i) but also higher catalytic activity for Li_2_S decomposition with a lower energy barrier (Fig. 6j). Therefore, the cell with Fe-N_2_/CN@S composite cathode exhibited a low capacity fading rate of only 0.011% per cycle after 2000 cycles at 2C (Fig. 6k). Co atom-doped g-C_3_N_4_ (Co@C_3_N_4_) material was also reported with a similar working mechanism in Li–S batteries [87]. The formation of Co-S bonds effectively immobilizes LiPSs.

Chen et al. compared the adsorption and electrocatalytic capability of single-metal-atom-doped g-C_3_N_4_ (M-C_3_N_4_, where M = Fe, Co, or Ni) for LiPSs [86]. According to DFT calculation results, the metal atoms’ doping can enhance the conductivity of g-C_3_N_4_, among which Fe-C_3_N_4_ and Co-C_3_N_4_ show semi-metallic properties, while Ni-C_3_N_4_ exhibits metallic properties. Moreover, as shown in Fig. 6l, Ni-C_3_N_4_ shows the strongest interaction with Li_2_S_6_ and leads to the largest response current when applied in Li–S batteries (Fig. 6m). In addition, Li_2_S decomposition presents the lowest energy barrier on the surface of the Ni-C_3_N_4_ substrate (Fig. 6n), suggesting that Ni-C_3_N_4_ can promote solid–liquid conversion. Therefore, the cell with a Ni-C_3_N_4_/C-modified separator performed a high capacity of 893 mAh g^−1^ after 300 cycles at 0.5 A g^−1^ with a capacity retention of 89.4%, showing good cycling stability and high S utilization efficiency (Fig. 6o).

To summarize, defect engineering and heteroatom doping are effective methods to regulate the adsorption capability and catalytic activity of g-C_3_N_4_. The performances of Li–S cells with optimized g-C_3_N_4_ materials are compared and listed in Table 1. However, excessive defects and heteroatoms could destroy the structure of g-C_3_N_4_. An in-depth understanding of the doping- and defect-structure–activity relationship still remains a grand challenge.

**Table 1:** Comparison of performances of Li–S cells with optimized g-C_3_N_4_ materials

Materials	S loading (mg cm^−2^)	S content (wt%)	Current rate (C)	Capacity (mAh g^−1^)/cycle number	Capacity decay rate (%)	References
Defective g-C_3_N_4_/CNTs	–	58.6	1	567/500	0.066	[78]
Defective g-C_3_N_4_/PDA	2	62.3	5	476/500	0.05	[58]
O-g-C_3_N_4_	–	39.2	0.5	447/500	0.1	[80]
B-g-C_3_N_4_	–	–	1	553/500	0.09	[81]
P-g-C_3_N_4_	1.5		0.2	882/100	0.34	[83]
Ni-g-C_3_N_4_/crystalline carbon	2.8	–	~ 0.3	893/300	0.035	[86]
Co@C_3_N_4_	2	–	~ 0.448	1160/200	0.086	[87]
Fe-g-C_3_N_4_	1.3–1.5	48.7	2	620/2000	0.011	[85]

## Design of g-C_3_N_4_-Based Composites

Although g-C_3_N_4_ has made remarkable progress in Li–S systems, it is difficult for pristine g-C_3_N_4_ to enable practical performance in Li–S batteries, owing to its intrinsic properties, including poor conductivity and low electrocatalytic activity. To explore novel g-C_3_N_4_-based materials with satisfying physical/chemical properties, g-C_3_N_4_ has been incorporated with other functional materials, such as conductive carbon materials, metal nanoparticles, and polar compounds. And the final g-C_3_N_4_-based composites exhibit various advantages, such as strong LiPSs immobilization, rapid Li-ion transfer, and accelerated conversion of S species.

### Conductive Carbon/g-C_3_N_4_ Composites

Various conductive carbon materials were applied in g-C_3_N_4_-based composite materials, including carbon nanotubes (CNTs), porous carbon material, graphene, and carbon cloth. The combination of g-C_3_N_4_ with conductive carbon materials could effectively improve the electronic conductivity of composite materials, which is critical for its application in Li–S batteries. In addition to this, different carbon materials could also provide various other benefits due to their specific structures and properties. For example, a large specific surface area of carbon materials could suppress LiPSs diffusion.

#### CNTs Constructed Conductive Networks

**Fig. 7:**
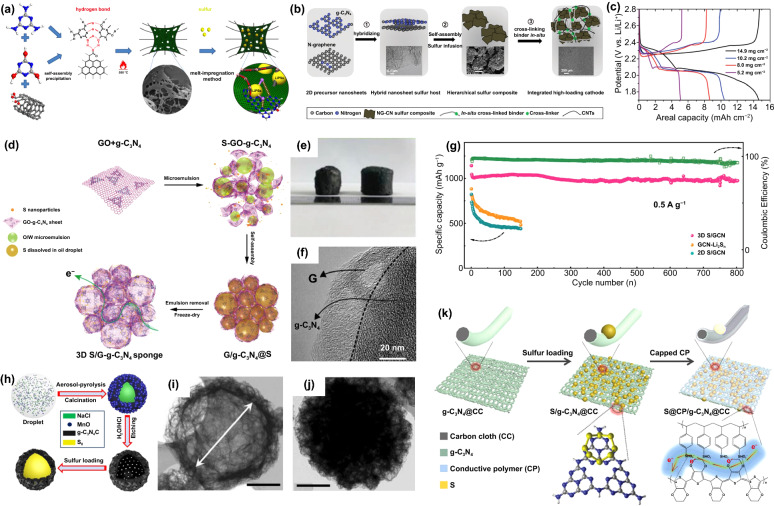
**a** Schematic illustration of the preparation process of S/CN-CNTs composite [33]. Copyright 2019, American Chemical Society. **b** Schematic illustration of the preparation process of NG-CN/CMC-CA/S composite. **c** Charge–discharge voltage profiles of cells using NG-CN/CMC-CA/S cathodes with different sulfur loadings at 0.5 mA cm^−2^ [42]. Copyright 2016, Wiley–VCH. **d** Schematic illustration of the preparation process of S/GCN hybrid sponge. **e** Optical images of G-sponge (left) and S/GCN hybrid sponge (right). **f** TEM image of S/GCN. **g** Cycling performance of 3D S/GCN, GCN-Li_2_S_n_ and 2D S-GCN cathodes at 0.5 A g^−1^ [32]. Copyright 2018, Wiley–VCH. **h** Schematic illustration of the preparation process of the S@C_3_N_4_/C microsphere. TEM images of **i** PC-C_3_N_4_/C and **j** S@C_3_N_4_/C [88]. Copyright 2020, Elsevier. **k** Schematic illustration of the preparation process of S@CP/g-C_3_N_4_@CC composite material [41]. Copyright 2020, Wiley–VCH

With the characteristic 1D structure and excellent electronic conductivity, CNTs could construct a conductive network and achieve fast electron conduction. By the high-temperature-assisted self-assembly method, Wang et al. directly synthesized g-C_3_N_4_ on the CNTs (Fig. 7a) [33]. Through hydrogen bonds, cyanic acid and melamine not only construct the triazine structure of g-C_3_N_4_ but also connect together with the CNTs. After heating treatment, the supramolecular structure can be further transformed into the final g-C_3_N_4_/CNTs composite. The obtained g-C_3_N_4_/CNTs composite shows a network structure with uniform coverage of the g-C_3_N_4_ layer. With largely improved conductivity and high LiPSs adsorption capability, the Li–S cell with g-C_3_N_4_/CNTs/S composite cathode displayed a high-capacity retention of 77.1% after 200 cycles at 1C at a 5 mg cm^−2^. Chen et al. [89] and Yao et al. [90] prepared g-C_3_N_4_/CNTs composite-based membranes and separately applied them as shielding layer and self-supported cathode. Profited from the strong LiPSs adsorption capability of g-C_3_N_4_ and good conductivity network of CNTs, the shuttle effect is largely inhibited, and S utilization efficiency is obviously improved.

#### Highly Conductive Graphene

Graphene is one of the most widely used materials to combine with g-C_3_N_4_. It processes a similar 2D honeycomb structure with g-C_3_N_4_ yet provides a much higher electronic conductivity even compared with CNTs. The obtained graphene/g-C_3_N_4_ composite shows increased conductivity than the original g-C_3_N_4_, which further leads to an enhanced cell performance when applied in Li–S systems. Based on the cohesion action between g-C_3_N_4_ precursor (melamine) and graphene oxide (GO), Nazar et al. synthesized a hybrid material (NG-CN) with high conductivity (Fig. 7b) [42]. And they subsequently coupled NG-CN with cellulose and citric acid (CMC-CA) to construct a stable composite cathode material (NG-CN/CMC-CA/S) with high S loading. The cell with NG-CN/CMC-CA/S composite cathode exhibited a high areal capacity of 14.7 mAh cm^−2^ with a sulfur loading of 14.9 mg cm^−2^, indicating the high S utilization efficiency (Fig. 7c). Besides, Dai et al. coated a 2D-layered composite material composed of graphene and g-C_3_N_4_ (C_3_N_4_/GS) on the S cathode surface [91]. Graphene as the upper collector accelerates the electron transfer in the cathode, and the strong adsorption capability of g-C_3_N_4_ inhibits the LiPSs diffusion. In addition, Guo et al. prepared a 3D porous S/graphene/g-C_3_N_4_ composite (S/GCN) with high conductivity and high stability by a microemulsion-assisted assembly method [32]. As shown in Fig. 7d, the internal oil emulsion dissolved sublimed S and acted as a soft template to create pores in the composite material, and the hydrophilic GCN tightly packed around the oil emulsion, thus forming a crosslinked 3D network structure. As the emulsion evaporates, the S attached evenly to the GCN walls and was eventually encased into the composite. The obtained S/GCN performs a cylindrical shape with graphene closely stacked with g-C_3_N_4_ nanosheets (Fig. 7e-f). The abundant N atoms and porous cross-linking network in GCN effectively limit the LiPSs dissolution and diffusion into the electrolyte. In addition, the 3D interconnected graphene network facilitates rapid electron transfer and maintains the integrity of the electrode structure, thus ensuring the long-term cycling stability of Li–S cells. The cell with S/GCN composite cathode displayed a high capacity of 612 mAh g^−1^ at 10C and maintained 974 mAh g^−1^ after 800 cycles at 0.5 A g^−1^ with a high capacity retention of 86% (Fig. 7g). Furthermore, by constructing heterostructures, the graphene/g-C_3_N_4_ composite could further achieve high electrocatalytic activity. Wang et al. used a phenyl modification strategy to construct g-C_3_N_4_/carbon heterostructures in situ on graphene sheets and coated them on the Celgard separator (G@g-C_3_N_4_/C) to inhibit the shuttle effect of LiPSs [92]. The g-C_3_N_4_/C heterostructure exhibits a unique electron distribution, showing a strong adsorption capability of LiPSs and high electrocatalytic activity for redox reactions. Therefore, the cell with G@g-C_3_N_4_/C coating revealed a low capacity fading rate of 0.050% per cycle after 800 cycles at 1 C.

#### Porous Carbon Materials Facilitated LiPSs Adsorption and Stable Structure

Porous carbon materials provide not only outstanding electronic conductivity but also controllable porosity and high specific surface area, which could facilitate the LiPSs adsorption. Qiu et al. reported g-C_3_N_4_ nanodots embedded MOF-derived N, S co-doped hollow porous carbon shells (CN@NSHPC) through a dual-solvent strategy [93]. Adsorption experiments show that the adsorption capability of CN@NSHPC composite for LiPSs is significantly higher than that of pristine g-C_3_N_4_ and NSHPC. Therefore, the cell using CN@NSHPC/S composite cathode displayed a low capacity fading rate of only 0.048% per cycle after 500 cycles at 1C, showing good cycling stability.

Besides, the porous carbon/g-C_3_N_4_ composite was also applied to construct the core–shell structure with S_8_ (Fig. 7h) [88]. As a shell, porous carbon/g-C_3_N_4_ composite provides high LiPSs adsorption, high electronic and ionic conductivity, and also enough space to alleviate the volume change of cathode during cycling [20]. The TEM images in Fig. 7i-j show that the g-C_3_N_4_/C composite performs a hollow spherical structure; after S loading, the cavity is filled to form a typical core–shell structure (S@g-C_3_N_4_/C). The porous g-C_3_N_4_/C shell promotes rapid electron transport and acts as a physical barrier in combination with chemisorption (abundant N atoms in g-C_3_N_4_) to synergistically inhibit LiPSs diffusion. Beyond that, Mandal et al. designed a double-shell structure, which is composed of a hollow mesoporous carbon (HCS) inner layer and a g-C_3_N_4_ outer layer [94]. The inner HCS layer provides high electron conductivity, high physical adsorption of LiPSs, and large spaces to mitigate the volume change of the electrode. The outer g-C_3_N_4_ shell can chemically anchor the LiPSs by forming Li-N bonds. Thus, the cell with HCS@g-C_3_N_4_/S composite cathode delivered a low capacity fading rate of 0.049% per cycle after 500 cycles at 1C.

#### Carbon Cloth Constructed Independent Electrode

The carbon cloth (CC) combined with g-C_3_N_4_ can not only enhance the electron conductivity of composites and can be used as an independent free-standing electrode. Without any current collector or binders, the energy density of batteries could be enhanced. Liu et al. prepared a g-C_3_N_4_/CC composite by *in situ* growing g-C_3_N_4_ nanosheets on the surface of 3D CC with abundant pores and used it as the S host [95]. Benefited from the strong LiPSs adsorption capability of g-C_3_N_4_ and the 3D conductive network of CC, the g-C_3_N_4_/CC/S composite cathode displayed good cycle stability with a high capacity of 892 mAh g^−1^ after 250 cycles at 0.2 C. Zheng et al. loaded poly (3, 4-ethylene dioxythiophene) (PEDOT) conductive polymer and g-C_3_N_4_ on commercial CC, and combined with S to form a free-standing, flexible cathode [41]. As shown in Fig. 7 k, g-C_3_N_4_@CC is synthesized by annealing CC with g-C_3_N_4_ precursor (urea) at high temperature, and S is introduced into g-C_3_N_4_@CC by sulfur–amine chemistry method. In order to further inhibit the LiPSs diffusion and improve the overall electrical conductivity of the electrode, PEDOT conductive polymer (CP) was introduced into the composite cathode by pressure impregnation, forming the S@CP/g-C_3_N_4_@CC. Thus, the cell with S@CP/g-C_3_N_4_@CC cathode exhibits a reversible capacity of 516.9 mAh g^−1^ after 500 cycles at 1C.

### Metal Nanoparticles/g-C_3_N_4_ Composites

**Fig. 8:**
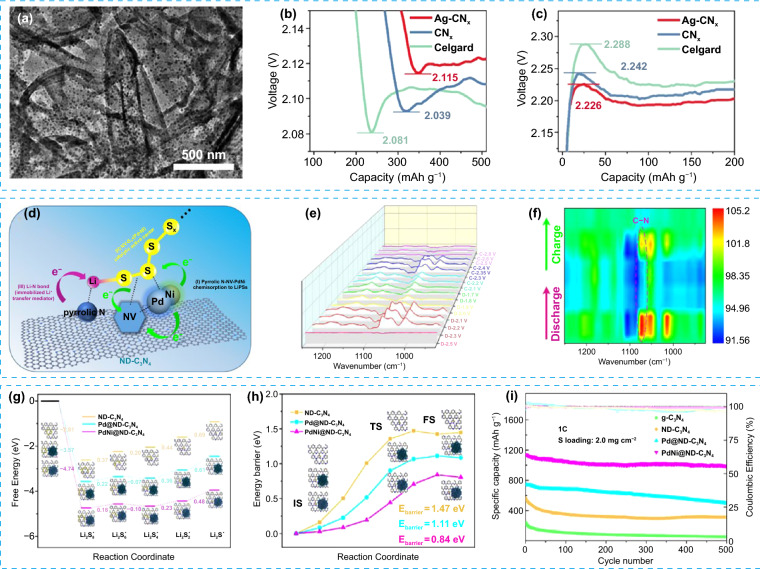
**a** TEM image of Ag-CN_x_. **b** Discharge profiles and **c** charge profiles of cells with Celgard separator, CN_x_ and Ag-CN_x_ modified separators [96]. Copyright 2021, Elsevier. **d** Schematic illustration of the interaction between PdNi@ND-C_3_N_4_ and LiPSs. **e** *In situ* FT-IR spectra of PdNi@ND-C_3_N_4_/S composite cathode at different discharge and charge stages, and **f** corresponding contour and response surface analysis. **g** The Gibbs free energies for the LiPSs reduction process on the ND-C_3_N_4_, Pd@ND-C_3_N_4_ and PdNi@ND-C_3_N_4_ substrates. **h** The energy barrier of Li_2_S decomposition on the ND-C_3_N_4_, Pd@ND-C_3_N_4_ and PdNi@ND-C_3_N_4_ substrates. **i** Cycling performance of cells with various S cathodes at 1C [97]. Copyright 2021, Elsevier

The introduction of metal nanoparticles into g-C_3_N_4_ could improve the electrical conductivity and adsorption/catalytic active sites of composites and further enhance the performance of Li–S batteries. Zhang et al. prepared Ag nanoparticles modified defective g-C_3_N_4_ (Ag-CN_x_) by the magnesium thermal reduction and "silver mirror" reaction [96]. The TEM image (Fig. 8a) shows that Ag particles are evenly distributed on the defective g-C_3_N_4_ nanosheets without agglomeration. As shown in Fig. 8b-c, the cell with Ag-CN_x_ modified separator shows the lowest energy barrier of the nucleation and dissolution reaction of Li_2_S. Thus, the modified cell exhibits outstanding cycling stability over 550 cycles at 2C. The Co nanoparticle-modified g-C_3_N_4_ composites also show high catalytic activity for LiPSs redox reactions, which were reported as functional materials for modified cathodes [98] and separators [99].

Compared with the strategy using a single kind of metal nanoparticles, the synergistic interaction between different kinds of metal nanoparticles can enhance the adsorption capability and electrocatalysis of g-C_3_N_4_ to LiPSs more effectively. Guo et al. prepared an electrocatalyst composed of a highly conductive N-deficient g-C_3_N_4_ (ND-C_3_N_4_) and a very small amount of hollow PdNi alloy nanospheres (PdNi@ND-C_3_N_4_) by using the galvanic substitution effect [97]. As shown in Fig. 8d, in the designed PdNi@ND-C_3_N_4_ composite, the PdNi alloy and introduced N vacancies exhibit strong chemisorption capability to LiPSs and high catalytic activity for their redox conversion. Moreover, the pyrrole ring of g-C_3_N_4_ provides a mediator for the rapid transfer of Li-ion with high lithiophilicity. In situ Fourier transform infrared spectroscopy (FT-IR) revealed the state changes of S species on the PdNi@ND-C_3_N_4_ surface during the charge and discharge process, as shown in Fig. 8e-f. When discharged to 2.3 V, soluble LiPSs were detected. The characteristic peak at 1073 cm^−1^ is related to the stretching vibration of asymmetric C-N bonds. The interaction between Li in LiPSs and electron-rich pyrrole rings in PdNi@ND-C_3_N_4_ causes the vibration of C-N bonds. With the increase of discharge depth, the strength of the C-N bond becomes weaker, indicating the conversion of soluble LiPSs to insoluble Li_2_S. When the discharge voltage reaches 2.0 V, the characteristic peak of the C-N bond disappears, indicating the completed conversion to Li_2_S at the cooperative catalytic interface of PdNi@ND-C_3_N_4_. In the charging process, the characteristic peak of the C-N bond appears first and then disappears, which demonstrates the Li_2_S is oxidized to soluble LiPSs and finally to S. The DFT calculation results in Fig. 8g-h show that compared with g-C_3_N_4_ materials combining one single-metal Pd, the reduction of S species and decomposition of Li_2_S at the surface of PdNi@ND-C_3_N_4_ showed a much lower Gibbs free energy change, indicating the synergistic catalysis of PdNi@ND-C_3_N_4_. Thus, the cell with PdNi@ND-C_3_N_4_/S composite cathode delivers a high discharge capacity of 989 mAh g^−1^ after 500 cycles at 1C (Fig. 8i); increasing the sulfur loading to 6.0 mg cm^−2^, the cell exhibits a low capacity fading rate of only 0.025% per cycle, suggesting the excellent cycling stability. Fe/Co-based g-C_3_N_4_/carbon composite material (Fe/Co-C_3_N_4_/C) shows similar synergistic catalysis [100]. With high conductivity, large specific surface area, and high catalytic activity, the cell with Fe/Co-C_3_N_4_/C/S composite cathode shows much enhanced electrochemical performance compared with that of C_3_N_4_/C/S cathode.

### Polar Compounds/g-C_3_N_4_ Composites

Polar compound, such as transition metal compound, is another representative material to composite with g-C_3_N_4_ due to their adsorption capability and catalytic activity. The strongly polar sites of polar compounds could interact with LiPSs and lower the energy barrier of their following reactions. Deng et al. uniformly dispersed lamellar CoS onto g-C_3_N_4_ nanosheets and then compounded them with conductive carbon (Ketjen black, KB) to prepare an ultra-thin multifunctional separator coating (CoS@g-C_3_N_4_/KB, ~ 2.1 μm) [103]. The Li-N bond and the Lewis acid–base interaction between CoS and LiPSs inhibit the shuttle effect. Metal oxide-based g-C_3_N_4_ composites, including TiO_2_/g-C_3_N_4_ [104] and Fe_3_O_4_/t-C_3_N_4_ [105], are also applied in Li–S batteries to improve S utilization and capacity retention.

**Fig. 9:**
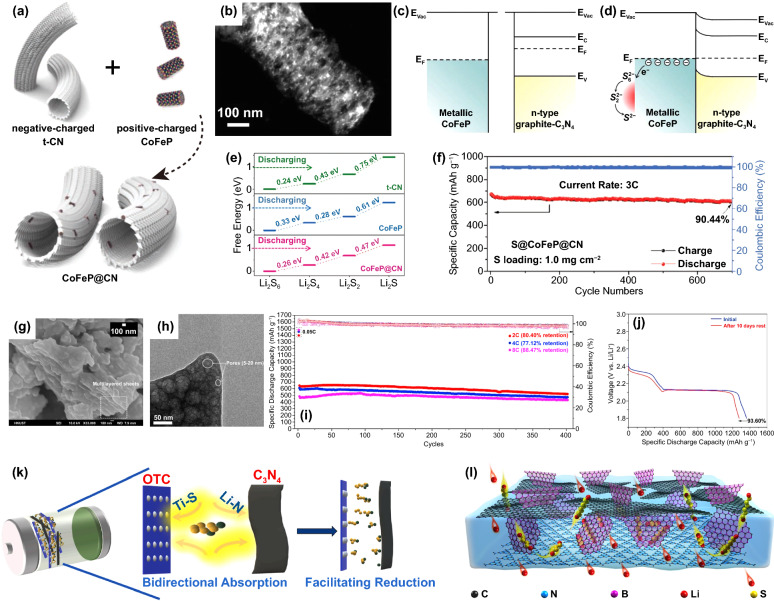
**a** Schematic illustration of the preparation process of CoFeP@CN composite. **b** TEM image of CoFeP@CN. Energy band diagrams of CoFeP and g-C_3_N_4_ before **c** and after **d** Schottky contact formation. **e** Gibbs free energies of the reduction reactions of Li_2_S_6_, Li_2_S_4_, Li_2_S_2_ and Li_2_S on t-CN, CoFeP and CoFeP@CN, respectively. **f** Cycling performance of the cell with S@CoFeP@CN cathode at 3C [101]. Copyright 2021, Wiley–VCH. **g** SEM image and **h** HRTEM image of MoS_2_/g-C_3_N_4_. **i** Cycling performance of the cell with MoS_2_/g-C_3_N_4_/S cathode at 2C, 4C and 8C. **j** Discharge voltage profiles showing the self-discharge behavior of MoS_2_/g-C_3_N_4_/S cathode [59]. Copyright 2019, Elsevier. **k** Schematic illustration of interaction mechanism of OTC/C_3_N_4_ and LiPSs [102]. Copyright 2021, Elsevier. **l** Schematic illustration of multifunctional ion-sieve composed of g-C_3_N_4_, BN, and graphene [52]. Copyright 2019, American Chemical Society

In addition to their own adsorption and catalytic capabilities, some compounds can also form heterostructures by composing with g-C_3_N_4,_ which would further improve their catalytic performance for redox conversion of S species. On the interfaces of heterostructures, electrons can be rearranged to modify the active sites, and the synergy of different active sites can promote reaction kinetics. By combing bimetallic phosphide CoFeP with g-C_3_N_4_ nanotube (t-CN), a Mott–Schottky heterojunction catalyst (CoFeP@CN) was prepared by Cabot et al. [101]. The tubular morphology of t-CN was maintained with CoFeP nanocrystals uniform distributed on the surface (Fig. 9a-b). As an n-type semiconductor, g-C_3_N_4_ has a work function of about 4.4 eV and a band gap of 2.6 eV, while the work function of CoFeP is about 4.8 eV (Fig. 9c). When they contact, the difference in Fermi energy levels drives electrons from g-C_3_N_4_ to CoFeP until their work functions reach equilibrium at the interface (Fig. 9d). In equilibrium, the electron band of g-C_3_N_4_ at the interface is bent upward, forming a Mott-Schottky heterostructure. CoFeP@CN has suitable electronic structure and charge rearrangement characteristics, which can accelerate the redox conversion of S species. Compared with CoFeP and t-CN, CoFeP@CN shows the lowest Gibbs free energy change, suggesting that CoFeP@CN heterojunction catalyst can promote the nucleation of Li_2_S (Fig. 9e). Moreover, the tubular shape of CoFeP@CN facilitates the diffusion of Li ions, alleviates volume changes of S cathode, and provides rich adsorption sites to effectively capture soluble LiPSs. Thus, the cell with S@CoFeP@CN composite cathode outputted a high capacity of 606 mAh g^−1^ after 700 cycles at 3C with a low capacity fading rate of 0.014% per cycle and a high coulombic efficiency of 99.6% (Fig. 9f). Chen et al. prepared another heterojunction composite MoS_2_/g-C_3_N_4_, with MoS_2_ nanosheets growing in situ on porous g-C_3_N_4_ nanosheets [59]. TEM images in Fig. 9g-h show that the MoS_2_/g-C_3_N_4_ composite has a lamellar and porous structure with a pore size of 5–20 nm. With strong chemical polarity, high porosity, and heterostructure, MoS_2_/g-C_3_N_4_ can effectively restrict the LiPSs diffusion and accelerate the redox conversion of S species. The cell with S/MoS_2_/g-C_3_N_4_ composite cathode delivered a high capacity retention of 88.47% after 400 cycles at 8C (Fig. 9i) and low self-discharge behavior of 0.026% per hour after 10 days (Fig. 9j), indicating the good rate capability and cycling stability.

With polar compounds/g-C_3_N_4_ composites, electrodes and separators with special structures were constructed, which further improved the performance of Li–S batteries. Huang et al. designed a sandwich cathode material (Fig. 9k), in which S was embedded between layered g-C_3_N_4_ and Ti_x_O_y_-Ti_3_C_2_ (OTC) [102]. The strong adsorption of g-C_3_N_4_ and Lewis acid–base interaction of the OTC heterojunction can immobilize LiPSs effectively. Furthermore, g-C_3_N_4_ and OTC simultaneously adsorb Li and S atoms in LiPSs, which can promote the cleavage of long-chain LiPSs and thus accelerate the reduction kinetics. The cell with such OTC/S/C_3_N_4_ cathode outputted a high discharge capacity of 750 mAh g^−1^ after 2000 cycles at 0.5C, showing outstanding cycling stability and high S utilization efficiency. Moreover, Dong et al. designed a multifunctional ion sieve composed of three kinds of 2D nanosheets, including g-C_3_N_4_, boron nitride (BN), and graphene (BN/graphene-C_3_N_4_), and used it as a separator coating [52]. As shown in Fig. 9l, the g-C_3_N_4_ overlaps with the graphene sheet to form a sandwich structure in which the BN sheet is vertically embedded. In the g-C_3_N_4_ monomer, there are ordered channels with a size of 3 Å, which can effectively prevent the LiPSs shuttling but allow free diffuse of Li ions; BN as a good electrocatalyst can accelerate the redox reaction of S species. The conductive network of graphene can promote the electron transport. Therefore, the cell with BN/graphene-C_3_N_4_ coating displayed a low capacity fading rate of 0.01% per cycle after 500 cycles at 1C with a high sulfur loading of 6 mg cm^−2^, suggesting the high S utilization efficiency.

The construction of g-C_3_N_4_-based composites offers new possibilities for g-C_3_N_4_ as additives in high-performance Li–S batteries. With different materials, the composites could realize the balance of various properties including catalytic activity and conductivity. The performances of Li–S cells with g-C_3_N_4_-based composites are compared and listed in Table 2. However, we note that most of the polar compounds were not combined with g-C_3_N_4_ alone but with conductive carbon materials at the same time [106]. Since the polar compounds applied show low conductivity as well as g-C_3_N_4_, carbon materials are required to construct a conductive network which enables the better catalytic performance of the materials. This leads to a notable issue of the increasing weight of inactive material in Li–S batteries.

**Table 2:** Comparison of performances of Li–S cells with g-C_3_N_4_-based composites

Materials	S loading (mg cm^−2^)	S content (wt%)	Current rate (C)	Capacity (mAh g^−1^)/cycle number	Capacity decay rate (%)	References
g-C_3_N_4_/Graphene oxide	–	–	~ 0.197	700/300	0.037	[107]
N-doped graphene- g-C_3_N_4_/cellulose-citric acid	2.0	65.45	0.05	–	–	[42]
g-C_3_N_4_@N, S co-doped hollow porous carbon shell	1.7–2.0	–	1	445/500	0.048	[93]
PEDOT/g-C_3_N_4_@CC	4.7	–	1	517/500	–	[41]
g-C_3_N_4_@porous carbon nanofiber	1.2	–	0.6	466/500	0.056	[108]
3D Graphene oxide-g-C_3_N_4_ sponge	4	73	~ 0.3	974/800	0.017	[32]
g-C_3_N_4_/carbon nanotubes	1	64	1	584/500	0.08	[33]
Reduced graphene oxide/ g-C_3_N_4_/carbon nanotubes	1.5	56.64	1	620/500	0.03	[34]
Hollow porous carbon nanosphere/g-C_3_N_4_	1	51.62	1	719/500	0.049	[94]
Hierarchical porous carbon/g-C_3_N_4_	1.0–1.2	51.6	1	757/250	0.024	[109]
g-C_3_N_4_/carbon spheres	1.2	46.9	1	636/400	0.09	[88]
g-C_3_N_4_/carbon nanotubes	4.74	–	0.5	633/300	0.092	[90]
g-C_3_N_4_/carbon cloth	2.5	–	0.2	892/250	–	[95]
g-C_3_N_4_/carbon fiber paper	2.21–2.66	45.06	–	–/400	0.068	[110]
Reduced graphene oxide /g-C_3_N_4_/carbon fiber paper	1.62–1.89	45	1	–/800	0.056	[111]
g-C_3_N_4_/graphene oxide	0.9–1.1	45.81	1	612/1000	–	[91]
graphene@g-C_3_N_4_/C	1.0	80	1	464/800	0.050	[92]
g-C_3_N_4_/carbon nanotubes	1.4	57.6	1	–/500	0.03	[89]
Co-g-C_3_N_4_	1.31	51.2	1	740/300	0.03	[98]
Ag-defective g-C_3_N_4_	1.3	53.3	2	652/550	0.053	[96]
Co-CNTs/defective g-C_3_N_4_	–	–	1	–/1000	0.04	[99]
PdNi@ND-C_3_N_4_	2	55.6	1	989/500	0.027	[97]
Fe/Co-C_3_N_4_/C	1.8	47.6	0.2	749/135	0.156	[100]
TiO_2_/g-C_3_N_4_	3.1	59.6	0.5	540/500	0.063	[104]
OTC/C_3_N_4_	1.2	–	0.5	750/2000	0.022	[102]
CoFeP@CN	1.0	–	3	606/700	0.014	[101]
MoS_2_/g-C_3_N_4_	1.5	59.1	2	521/400	0.049	[59]
CoS@g-C_3_N_4_/KB	1.5	68.4	1	572/500	0.030	[103]
Fe_3_O_4_/t-C_3_N_4_	0.8–1.0	–	2	658/1000	0.020	[105]
BN/graphene-C_3_N_4_	6.0	70	1	603/500	0.01	[52]

## Other C_*x*_N_*y*_ Materials

With different C/N ratios, carbon nitride (C_x_N_y_), other than g-C_3_N_4_, shows different molecular configurations, which endows them with different physicochemical characteristics and electronic properties.

**Fig. 10:**
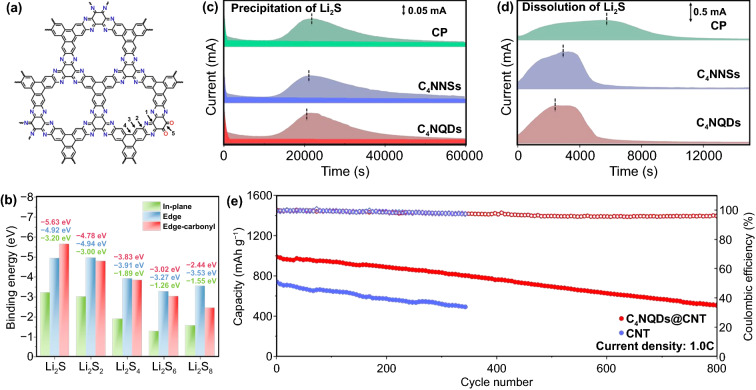
**a** Schematic illustration of the structure of C_4_N. **b** Adsorption energies for Li_2_S, Li_2_S_2_, Li_2_S_4_, Li_2_S_6_, and Li_2_S_8_ on various sites of C_4_N. **c** Potentiostatic discharge curves and **d** potentiostatic charge curves of Li_2_S precipitation on the CP, C_4_NNSs/CP and C_4_NQDs/CP. **e** Cycling performance of cells with CNT modified separator and C_4_NQDs@CNT modified separator at 1.0C [112]. Copyright 2021, Wiley–VCH

The regulation of the C/N ratio could change the coordination environment of N, which further leads to enhanced catalytic performance. Yu et al. reported a covalent organic framework (COF)-like C_x_N_y_ material, C_4_N, with a C/N ratio of 4:1 connected with pyrazine, and subsequently prepared ultra-small colloidal C_4_N quantum dots (C_4_NQDs) with the average size of 2.2 nm (Fig. 10a) [112]. Pyrazine N atoms and carbonyl groups at the edge of C_4_NQDs show preferential adsorption capability to LiPSs with significantly higher binding energy compared with that of the N sites in the C_4_NQDs plane (Fig. 10b). The electrochemical performance further confirmed the excellent catalytic capability of C_4_NQDs for LiPSs redox reactions (Fig. 10c-d). Compared with that on pristine carbon paper (CP), the Li_2_S precipitation and decomposition on C_4_NQDs/CP perform higher capacity contributions and an earlier peak current response. Therefore, the Li–S cell with C_4_NQDs@CNTs modified separator exhibited excellent cycling stability with a low capacity fading rate of 0.061% per cycle after 800 cycles at 1C (Fig. 10e). Besides, the LiPSs adsorption on 2 D C_2_N nanosheets was investigated by Wang et al. [113]. Unlike g-C_3_N_4,_ C_2_N is connected by one N atom and two C atoms with higher structural stability. The original graphene C-ring structure is surrounded by six N atoms in C_2_N, each of which has a suspended bond. The size of the hole formed in the middle is smaller than that of long-chain LiPSs, which could physically inhibit the shuttle effect. In addition, C_2_N shows highly negative Gibbs free energy for Li_n_-C_2_N and strong adsorption capability for Li_2_S. Its high LiPSs adsorption capability was also confirmed by Zhang et al. using first-principles calculations [114]. Comparing four nonmetallic layered materials (graphene, BN, C_2_N, and C_3_N_4_), C_3_N_4_ and C_2_N exhibit stronger LiPSs adsorption capability through the interfacial interactions and inhibit the dissolution of LiPSs into the electrolyte.

It is worth noting that, in addition to the adsorption and catalytic capability, the electron conductivity of the materials can also be regulated with different C/N ratios. Zhang et al. designed a new 2D carbon nitride, C_5_N, by introducing the vacancy defects into monolayer C_3_N and studied its adsorption capability for LiPSs [115]. After the introduction of V_C_ + V_N_ vacancy defects in C_3_N and the optimization of the full structure, the new configuration is composed of 5 carbon nitrogen ring-8 carbon nitrogen ring-5 carbon ring. The electronic band structure and state density (DOS) reveal that the Fermi level of C_5_N is below the maximum value of the valence band and its band gap structure is similar to that of Cu metal, indicating that C_5_N has good electrical conductivity and metallic properties. Besides, it also shows an adsorption capability of both LiPSs and Li_2_S. Comparing 10 kinds of N-containing 2D materials (C_9_N_4_, C_2_N, BN, CTF, C_2_N_6_S_3_, g-C_3_N_4_, p-C_3_N_4_, C_3_N_5_, S-N_2_S, and T-N_2_S), Chen et al. found that the C_9_N_4_ and C_2_N_6_S_3_ perform good electronic conductivity and strong adsorption for LiPSs [116]. With increased electronic conductivity of C_x_N_y_, the LiPSs adsorbed can directly lose/gain electrons to be oxidized/reduced, which avoids the loss of LiPSs and improve the rate capability of Li–S batteries.

However, other than g-C_3_N_4_, most of the C_x_N_y_ materials used in Li–S batteries have only been reported in simulation. Future experimental studies will benefit the understanding and application of these materials.

## Summary and Outlook

To date, C_x_N_y_ materials, represented by g-C_3_N_4_, have been widely applied in Li–S batteries as additives to enhance electrochemical performance due to their strong LiPSs adsorption capability and high tunability in composition and structure, which leads to controllable properties. With the advancing studies on C_x_N_y_-based materials, the structure–activity relationship is gradually revealed. The changes in the chemical composition, for example, the regulation of the C/N ratio and the introduction of heteroatoms, could modulate the coordination structure of catalytic active sites and the electronic structure of the material, leading to increased catalytic capability and electronic conductivity. The construction of various structures, such as porous nanosheet structure, spindle-like structure, and hollow spherical structure, could lead to high specific surface area and increasingly exposure of active sites, which also enhance the adsorption and electrocatalytic capability of C_x_N_y_ materials and further improve the rate performance of Li–S batteries. Besides, the hollow or core–shell structure could also accommodate the volume change during cycling and increase the cycle life of Li–S batteries. However, the understanding of the structure–activity relationship of C_x_N_y_ is still limited. In-depth understandings, such as the effects of doping and defect structures on the catalytic and conductive performance, are critical for the development of C_x_N_y_-based materials for advanced Li–S batteries.

At this stage, Li–S batteries are developing toward practicality. The demonstration for practical specific energy and stability dominates the future development direction of Li–S batteries. Based on the understanding of C_x_N_y_-based materials and Li–S chemistry, we identified the existing limitations and provided our perspective on future rational design of advanced C_x_N_y_-based materials for high-performance Li–S batteries:

1. As an inactive material, C_x_N_y_ should be added at low amounts to increase the overall energy density of the Li–S cell. According to some reported works [35, 45], g-C_3_N_4_ added as S host accounts for about 15% of the total cathode weight. The reduction of C_x_N_y_ content requires an increase in their specific surface area, which means an increased in the adsorption capability, and therefore, a similar function could be realized with a smaller amount of C_x_N_y_ materials. Different preparation methods, such as the hard template method and stripping-assisted method, can be used to obtain the g-C_3_N_4_ with controllable morphology and high specific surface area;
2. The electronic conductivity of g-C_3_N_4_, which limits the rate performance of Li–S batteries, should be increased. Although improved by numerous reported methods, the conductivity of g-C_3_N_4_ is still unsatisfactory. The effect of heteroatom doping and defect treatment on the conductivity of g-C_3_N_4_ appears limited; the additional carbon material introduced in the form of g-C_3_N_4_/carbon composite increases the amount of inactive materials. Besides these methods, the electronic conductivity of the materials could also be increased by regulating the C/N ratios. Synthesizing novel C_x_N_y_ materials with higher electron conductivity and studying their adsorption and electrocatalytic effect on LiPSs could be prospective directions in Li–S systems.
3. The catalytic mechanism of C_x_N_y_-based materials should be systematically studied. Although most of the C_x_N_y_-based materials are proposed with a working mechanism, the reaction mechanism of S_8_ with C_x_N_y_-based materials is still unclear. Since the conversion process of LiPSs is quite complex, it is necessary to combine advanced *in situ* characterization techniques such as cryo-electron microscopy, *in situ* Raman, and XRD to monitor the electronic structure and morphology changes of intermediates on C_x_N_y_-based materials under various conditions in real time. By comparing series of materials in parallel, the structure–activity relationship can be revealed, which is critical for further material design.
4. The enhancement of Li anode stability with C_x_N_y_-based materials worths more attention. The growth of dendrite limits the cycling life of Li anode. Besides, the soluble LiPSs lead to the passivation of Li anode in Li–S batteries. g-C_3_N_4_ has high shear modulus and good affinity with Li ions, and thus, it is promising in promoting the uniform deposition of Li ions and inhibiting the growth of Li dendrites [39, 40]. However, relevant studies are very limited. With more effort on this topic, an enhancement of Li anode stability with C_x_N_y_-based material is expected.

Looking into the future, there are infinite opportunities and challenges for the vigorous development of C_x_N_y_-based materials. With further efforts, it is expected that C_x_N_y_-based materials will promote the practical application of high energy density and long-life Li–S batteries.

## References

[1] Tushar W, Saha TK, Yuen C, Smith D, Poor HV (2020). Peer-to-peer trading in electricity networks: an overview. IEEE Trans. Smart Grid.

[2] Ahmad T, Zhang D (2020). A critical review of comparative global historical energy consumption and future demand: the story told so far. Energy Rep..

[3] Carley S, Konisky DM (2020). The justice and equity implications of the clean energy transition. Nat. Energy.

[4] Abdin Z, Zafaranloo A, Rafiee A, Mérida W, Lipiński W (2020). Hydrogen as an energy vector. Renew. Sustain. Energy Rev..

[5] Zhao L, Liu Z, Chen D, Liu F, Yang Z (2021). Laser synthesis and microfabrication of micro/nanostructured materials toward energy conversion and storage. Nano-Micro Lett..

[6] Wu F, Maier J, Yu Y (2020). Guidelines and trends for next-generation rechargeable lithium and lithium-ion batteries. Chem. Soc. Rev..

[7] Zhao S, Kang Y, Liu M, Wen B, Fang Q (2021). Modulating the electronic structure of nanomaterials to enhance polysulfides confinement for advanced lithium–sulfur batteries. J. Mater. Chem. A.

[8] He J, Manthiram A (2019). A review on the status and challenges of electrocatalysts in lithium–sulfur batteries. Energy Storage Mater..

[9] Hencz L, Chen H, Ling HY, Wang Y, Lai C (2019). Housing sulfur in polymer composite frameworks for Li-S batteries. Nano-Micro Lett..

[10] Li Y, Guo S (2021). Material design and structure optimization for rechargeable lithium–sulfur batteries. Matter.

[11] Deng W, Phung J, Li G, Wang X (2021). Realizing high-performance lithium–sulfur batteries via rational design and engineering strategies. Nano Energy.

[12] Deng C, Wang Z, Wang S, Yu J (2019). Inhibition of polysulfide diffusion in lithium–sulfur batteries: mechanism and improvement strategies. J. Mater. Chem. A.

[13] Hou L, Zhang X, Li B, Zhang Q (2021). Challenges and promises of lithium metal anode by soluble polysulfides in practical lithium–sulfur batteries. Mater. Today.

[14] Yin Y, Xin S, Guo Y, Wan L (2013). Lithium–sulfur batteries: electrochemistry, materials, and prospects. Angew. Chem. Int. Ed..

[15] Mikhaylik YV, Akridge JR (2004). Polysulfide shuttle study in the Li/S battery system. J. Electrochem. Soc..

[16] Zu C, Chung SH, Manthiram A (2015). Lithium–sulfur batteries: progress and prospects. Adv. Mater..

[17] P.P.R.M.L. Harks, C.B. Robledo, T.W. Verhallen, P.H.L. Notten, F.M. Mulder, The significance of elemental sulfur dissolution in liquid electrolyte lithium sulfur batteries. Adv. Energy Mater. **7**(3), 1601635 (2017). Doi: 10.1002/aenm.201601635

[18] Cheng XB, Huang JQ, Zhang Q (2017). Review—Li metal anode in working lithium–sulfur batteries. J. Electrochem. Soc..

[19] Zhou W, Yu Y, Chen H, DiSalvo FJ, Abruna HD (2013). Yolk-shell structure of polyaniline-coated sulfur for lithium–sulfur batteries. J. Am. Chem. Soc..

[20] Seh ZW, Li W, Cha JJ, Zheng G, Yang Y (2013). Sulphur-TiO_2_ yolk-shell nanoarchitecture with internal void space for long-cycle lithium-sulphur batteries. Nat. Commun..

[21] Kim J, Kim SJ, Jung E, Mok DH, Paidi VK (2022). Atomic structure modification of Fe-N-C catalysts via morphology engineering of graphene for enhanced conversion kinetics of lithium–sulfur batteries. Adv. Funct. Mater..

[22] Li Y, Wang W, Zhang B, Fu L, Wan M (2021). Manipulating redox kinetics of sulfur species using Mott–Schottky electrocatalysts for advanced lithium–sulfur batteries. Nano Lett..

[23] Li S, Fan Z (2021). Encapsulation methods of sulfur particles for lithium–sulfur batteries: a review. Energy Storage Mater..

[24] Zhang X, Wei Y, Wang B, Wang M, Zhang Y (2019). Construction of electrocatalytic and heat-resistant self-supporting electrodes for high-performance lithium–sulfur batteries. Nano-Micro Lett..

[25] Yang JL, Cai DQ, Lin Q, Wang XY, Fang ZQ (2022). Regulating the Li_2_S deposition by grain boundaries in metal nitrides for stable lithium–sulfur batteries. Nano Energy.

[26] Zhou C, Wang J, Zhu X, Chen K, Ouyang Y (2021). A dual-functional poly(vinyl alcohol)/poly(lithium acrylate) composite nanofiber separator for ionic shielding of polysulfides enables high-rate and ultra-stable Li-S batteries. Nano Res..

[27] Wang S, Liu X, Duan H, Deng Y, Chen G (2021). Fe_3_C/Fe nanoparticles embedded in N-doped porous carbon nanosheets and graphene: a thin functional interlayer for PP separator to boost performance of Li-S batteries. Chem. Eng. J..

[28] Wang J, Cai W, Mu X, Han L, Wu N (2021). Designing of multifunctional and flame retardant separator towards safer high-performance lithium–sulfur batteries. Nano Res..

[29] Zheng N, Jiang G, Chen X, Mao J, Jiang N (2019). Battery separators functionalized with edge-rich MoS_2_/C hollow microspheres for the uniform deposition of Li_2_S in high-performance lithium–sulfur batteries. Nano-Micro Lett..

[30] Zhan Y, Buffa A, Yu L, Xu ZJ, Mandler D (2020). Electrodeposited sulfur and Co_x_S electrocatalyst on buckypaper as high-performance cathode for Li-S batteries. Nano-Micro Lett..

[31] Jiang B, Tian D, Qiu Y, Song X, Zhang Y (2021). High-index faceted nanocrystals as highly efficient bifunctional electrocatalysts for high-performance lithium–sulfur batteries. Nano-Micro Lett..

[32] Zhang J, Li JY, Wang WP, Zhang XH, Tan XH (2018). Microemulsion assisted assembly of 3D porous S/graphene@g-C_3_N_4_ hybrid sponge as free-standing cathodes for high energy density Li–S batteries. Adv. Energy Mater..

[33] He W, He X, Du M, Bie S, Liu J (2019). Three-dimensional functionalized carbon nanotubes/graphitic carbon nitride hybrid composite as the sulfur host for high-performance lithium–sulfur batteries. J. Phys. Chem. C.

[34] Wang J, Meng Z, Yang W, Yan X, Guo R (2019). Facile synthesis of rGO/g-C_3_N_4_/CNT microspheres via an ethanol-assisted spray-drying method for high-performance lithium–sulfur batteries. ACS Appl. Mater. Interfaces.

[35] Pang Q, Nazar LF (2016). Long-life and high-areal-capacity Li–S batteries enabled by a light-weight polar host with intrinsic polysulfide adsorption. ACS Nano.

[36] Liao K, Mao P, Li N, Han M, Yi J (2016). Stabilization of polysulfides via lithium bonds for Li–S batteries. J. Mater. Chem. A.

[37] Wang Z, Jin B, Peng J, Su W, Zhang K (2021). Engineered polymeric carbon nitride additive for energy storage materials: a review. Adv. Funct. Mater..

[38] Adekoya D, Qian S, Gu X, Wen W, Li D (2020). DFT-guided design and fabrication of carbon-nitride-based materials for energy storage devices: a review. Nano-Micro Lett..

[39] Xiong C, Ren Y, Jiang H, Wu M, Zhao T (2019). Artificial bifunctional protective layer composed of carbon nitride nanosheets for high performance lithium–sulfur batteries. J. Energy Storage.

[40] Bai M, Hong B, Zhang K, Yuan K, Xie K (2021). Defect-rich carbon nitride as electrolyte additive for in-situ electrode interface modification in lithium metal battery. Chem. Eng. J..

[41] Li H, Chen H, Xue Y, Zhang Y, Zhang M (2020). Catalytic and dual-conductive matrix regulating the kinetic behaviors of polysulfides in flexible Li–S batteries. Adv. Energy Mater..

[42] Pang Q, Liang X, Kwok CY, Kulisch J, Nazar LF (2016). A comprehensive approach toward stable lithium–sulfur batteries with high volumetric energy density. Adv. Energy Mater..

[43] Yao S, Xue S, Peng S, Guo R, Wu Z (2018). Synthesis of graphitic carbon nitride via direct polymerization using different precursors and its application in lithium–sulfur batteries. Appl. Phys. A.

[44] D. Versaci, M. Cozzarin, J. Amici, C. Francia, E.P.M. Leiva et all., Influence of synthesis parameters on g-C_3_N_4_ polysulfides trapping: a systematic study. Appl. Mater. Today **25**, 101169 (2021). Doi: 10.1016/j.apmt.2021.101169

[45] Meng Z, Li S, Ying H, Xu X, Zhu X (2017). From silica sphere to hollow carbon nitride-based sphere: rational design of sulfur host with both chemisorption and physical confinement. Adv. Mater. Interfaces.

[46] Du M, Tian X, Ran R, Zhou W, Liao K (2020). Tuning nitrogen in graphitic carbon nitride enabling enhanced performance for polysulfide confinement in Li–S batteries. Energy Fuels.

[47] Wang X, Blechert S, Antonietti M (2012). Polymeric graphitic carbon nitride for heterogeneous photocatalysis. ACS Catal..

[48] Xue D, Xia H, Yan W, Zhang J, Mu S (2020). Defect engineering on carbon-based catalysts for electrocatalytic CO_2_ reduction. Nano-Micro Lett..

[49] S. Ghosh, S. Ramaprabhu, High-pressure investigation of ionic functionalized graphitic carbon nitride nanostructures for CO_2_ capture. J. CO_2_ Util. **21**, 89–99 (2017). Doi: 10.1016/j.jcou.2017.06.022

[50] Wang Z, Hu X, Liu Z, Zou G, Wang G (2019). Recent developments in polymeric carbon nitride-derived photocatalysts and electrocatalysts for nitrogen fixation. ACS Catal..

[51] Kessler FK, Zheng Y, Schwarz D, Merschjann C, Schnick W (2017). Functional carbon nitride materials-design strategies for electrochemical devices. Nat. Rev. Mater..

[52] Deng D, Bai C, Xue F, Lei J, Xu P (2019). Multifunctional ion-sieve constructed by 2D materials as an interlayer for Li–S batteries. ACS Appl. Mater. Interfaces.

[53] Wang Z, Jin B, Zou G, Zhang K, Hu X (2019). Rationally designed copper-modified polymeric carbon nitride as a photocathode for solar water splitting. Chemsuschem.

[54] Merschjann C, Tschierlei S, Tyborski T, Kailasam K, Orthmann S (2015). Complementing graphenes: 1D interplanar charge transport in polymeric graphitic carbon nitrides. Adv. Mater..

[55] Hou Y, Wen Z, Cui S, Guo X, Chen J (2013). Constructing 2D porous graphitic C_3_N_4_ nanosheets/nitrogen-doped graphene/layered MoS_2_ ternary nanojunction with enhanced photoelectrochemical activity. Adv. Mater..

[56] Wang Y, Wang X, Antonietti M (2012). Polymeric graphitic carbon nitride as a heterogeneous organocatalyst: from photochemistry to multipurpose catalysis to sustainable chemistry. Angew. Chem. Int. Ed..

[57] Li X, Xu C, Zhao K, Wang Y, Pan L (2016). Carbon nitride based mesoporous materials as cathode matrix for high performance lithium–sulfur batteries. RSC Adv..

[58] Tong Z, Huang L, Liu H, Lei W, Zhang H (2021). Defective graphitic carbon nitride modified separators with efficient polysulfide traps and catalytic sites for fast and reliable sulfur electrochemistry. Adv. Funct. Mater..

[59] Majumder S, Shao M, Deng Y, Chen G (2019). Ultrathin sheets of MoS_2_/g-C_3_N_4_ composite as a good hosting material of sulfur for lithium–sulfur batteries. J. Power Sources.

[60] Jia Z, Zhang H, Yu Y, Chen Y, Yan J (2020). Trithiocyanuric acid derived g-C_3_N_4_ for anchoring the polysulfide in Li–S batteries application. J. Energy Chem..

[61] Yao S, Xue S, Peng S, Jing M, Qian X (2018). Synthesis of graphitic carbon nitride at different thermal-pyrolysis temperature of urea and it application in lithium–sulfur batteries. J. Mater. Sci. Mater. Electron..

[62] Yang X (2019). Core-shell S@C_3_N_4_ nano-spheres as advanced adsorbent material for excellent lithium storage. Mater. Res. Express.

[63] Zhao F, Nani M, Kun Z, Keyu X, Chao S (2019). Handheld spraying of g-C_3_N_4_ nanosheets on cathode for high-performance lithium–sulfur batteries. Ionics.

[64] Huangfu Y, Zheng T, Zhang K, She X, Xu H (2018). Facile fabrication of permselective g-C_3_N_4_ separator for improved lithium–sulfur batteries. Electrochim. Acta.

[65] Yu H, Shi R, Zhao Y, Bian T, Zhao Y (2017). Alkali-assisted synthesis of nitrogen deficient graphitic carbon nitride with tunable band structures for efficient visible-light-driven hydrogen evolution. Adv. Mater..

[66] Zhao D, Dong CL, Wang B, Chen C, Huang YC (2019). Synergy of dopants and defects in graphitic carbon nitride with exceptionally modulated band structures for efficient photocatalytic oxygen evolution. Adv. Mater..

[67] Dong Y, Zhang Q, Tian Z, Li B, Yan W (2020). Ammonia thermal treatment toward topological defects in porous carbon for enhanced carbon dioxide electroreduction. Adv. Mater..

[68] Wang J, Han WQ (2021). A review of heteroatom doped materials for advanced lithium–sulfur batteries. Adv. Funct. Mater..

[69] Wang R, Wu R, Ding C, Chen Z, Xu H (2021). Porous carbon architecture assembled by cross-linked carbon leaves with implanted atomic cobalt for high-performance Li–S batteries. Nano-Micro Lett..

[70] Shi M, Zhang S, Jiang Y, Jiang Z, Zhang L (2020). Sandwiching sulfur into the dents between N, O co-doped graphene layered blocks with strong physicochemical confinements for stable and high-rate Li–S batteries. Nano-Micro Lett..

[71] Zhuang Z, Kang Q, Wang D, Li Y (2020). Single-atom catalysis enables long-life, high-energy lithium–sulfur batteries. Nano Res..

[72] Fang L, Feng Z, Cheng L, Winans RE, Li T (2020). Design principles of single atoms on carbons for lithium–sulfur batteries. Small Methods.

[73] Liu Z, Zhou L, Ge Q, Chen R, Ni M (2018). Atomic iron catalysis of polysulfide conversion in lithium–sulfur batteries. ACS Appl. Mater. Interfaces.

[74] Yang T, Liu K, Wu T, Zhang J, Zheng X (2020). Rational valence modulation of bimetallic carbide assisted by defect engineering to enhance polysulfide conversion for lithium–sulfur batteries. J. Mater. Chem. A.

[75] Shi Z, Li M, Sun J, Chen Z (2021). Defect engineering for expediting Li–S chemistry: strategies, mechanisms, and perspectives. Adv. Energy Mater..

[76] Zhang Y, Li G, Wang J, Cui G, Wei X (2020). Hierarchical defective Fe_3-x_C@C hollow microsphere enables fast and long-lasting lithium–sulfur batteries. Adv. Funct. Mater..

[77] He D, Meng J, Chen X, Liao Y, Cheng Z (2020). Ultrathin conductive interlayer with high-density antisite defects for advanced lithium–sulfur batteries. Adv. Funct. Mater..

[78] Ma H, Song C, Liu N, Zhao Y, Bakenov Z (2020). Nitrogen-deficient graphitic carbon nitride/carbon nanotube as polysulfide barrier of high-performance lithium–sulfur batteries. ChemElectroChem.

[79] Li D, Liu J, Wang W, Li S, Yang G (2021). Synthesis of porous n deficient graphitic carbon nitride and utilization in lithium–sulfur battery. Appl. Surf. Sci..

[80] Liu J, Li W, Duan L, Li X, Ji L (2015). A graphene-like oxygenated carbon nitride material for improved cycle-life lithium/sulfur batteries. Nano Lett..

[81] Jiang R, Jiang M, Huang Z, Wang J, Kuang Y (2020). Constructing light-weight polar boron-doped carbon nitride nanosheets with increased active sites and conductivity for high performance lithium–sulfur batteries. Int. J. Hydrog. Energy.

[82] Yamsang N, Sittiwong J, Srifa P, Boekfa B, Sawangphruk M (2021). First-principle study of lithium polysulfide adsorption on heteroatom doped graphitic carbon nitride for lithium–sulfur batteries. Appl. Surf. Sci..

[83] Zhang X, Yang S, Chen Y, Li S, Tang S (2020). Effect of phosphorous-doped graphitic carbon nitride on electrochemical properties of lithium–sulfur battery. Ionics.

[84] He F, Li K, Yin C, Ding Y, Tang H (2018). A combined theoretical and experimental study on the oxygenated graphitic carbon nitride as a promising sulfur host for lithium–sulfur batteries. J. Power Sources.

[85] Qiu Y, Fan L, Wang M, Yin X, Wu X (2020). Precise synthesis of Fe-N_2_ sites with high activity and stability for long-life lithium–sulfur batteries. ACS Nano.

[86] Chen M, Zhao X, Li Y, Zeng P, Liu H (2020). Kinetically elevated redox conversion of polysulfides of lithium–sulfur battery using a separator modified with transition metals coordinated g-C_3_N_4_ with carbon-conjugated. Chem. Eng. J..

[87] Wu J, Chen J, Huang Y, Feng K, Deng J (2019). Cobalt atoms dispersed on hierarchical carbon nitride support as the cathode electrocatalyst for high-performance lithium-polysulfide batteries. Sci. Bull..

[88] Song P, Chen Z, Chen Y, Ma Q, Xia X (2020). Light-weight g-C_3_N_4_/carbon hybrid cages as conductive and polar hosts to construct core-shell structured S@g-C_3_N_4_/carbon spheres with enhanced Li ion-storage performance. Electrochim. Acta.

[89] Wang X, Li G, Li M, Liu R, Li H (2021). Reinforced polysulfide barrier by g-C_3_N_4_/CNT composite towards superior lithium–sulfur batteries. J. Energy Chem..

[90] Wu Z, Yao S, Guo R, Li Y, Zhang C (2020). Freestanding graphitic carbon nitride-based carbon nanotubes hybrid membrane as electrode for lithium/polysulfides batteries. Int. J. Energy Res..

[91] Qu L, Liu P, Yi Y, Wang T, Yang P (2019). Enhanced cycling performance for lithium–sulfur batteries by a laminated 2D g-C_3_N_4_/graphene cathode interlayer. Chemsuschem.

[92] Zhang H, Liu Q, Ruan S, Ma C, Jia X (2022). In-situ construction of g-C_3_N_4_/carbon heterostructure on graphene nanosheet: an efficient polysulfide barrier for advanced lithium–sulfur batteries. Appl. Surf. Sci..

[93] Zhang H, Zhao Z, Hou YN, Tang Y, Dong Y (2018). Nanopore-confined g-C_3_N_4_ nanodots in N, S co-doped hollow porous carbon with boosted capacity for lithium–sulfur batteries. J. Mater. Chem. A.

[94] Ma J, Yu M, Ye H, Song H, Wang D (2019). A 2D/2D graphitic carbon nitride/N-doped graphene hybrid as an effective polysulfide mediator in lithium–sulfur batteries. Mater. Chem. Front..

[95] Zhang H, Lin X, Li J, Han T, Zhu M (2021). A binder-free lithium–sulfur battery cathode using three-dimensional porous g-C_3_N_4_ nanoflakes as sulfur host displaying high binding energies with lithium polysulfides. J. Alloys Compd..

[96] Zhang K, Cai W, Liu Y, Hu G, Hu W (2022). Nitrogen-doped carbon embedded with Ag nanoparticles for bidirectionally-promoted polysulfide redox electrochemistry. Chem. Eng. J..

[97] Zhou X, Liu T, Zhao G, Yang X, Guo H (2021). Cooperative catalytic interface accelerates redox kinetics of sulfur species for high-performance Li–S batteries. Energy Storage Mater..

[98] Wang M, Zhou X, Cai X, Wang H, Fang Y (2020). Hierarchically porous, ultrathin N–doped carbon nanosheets embedded with highly dispersed cobalt nanoparticles as efficient sulfur host for stable lithium–sulfur batteries. J. Energy Chem..

[99] Feng Y, Wang G, Wang L, Ju J, Kang W (2021). Taming polysulfides and facilitating redox: novel interlayer based on chestnut-like and multi-level structural materials for ultra-stable lithium–sulfur batteries. J. Alloys Compd..

[100] Li Y, Chen M, Zeng P, Liu H, Yu H (2021). Fe, Co-bimetallic doped C_3_N_4_ with in-situ derived carbon tube as sulfur host for anchoring and catalyzing polysulfides in lithium–sulfur battery. J. Alloys Compd..

[101] Zhang C, Du R, Biendicho JJ, Yi M, Xiao K (2021). Tubular CoFeP@CN as a Mott-Schottky catalyst with multiple adsorption sites for robust lithium−sulfur batteries. Adv. Energy Mater..

[102] Pan H, Huang X, Wang C, Liu D, Wang D (2021). Sandwich structural Ti_x_O_y_-Ti_3_C_2_/C_3_N_4_ material for long life and fast kinetics lithium–sulfur battery: bidirectional adsorption promoting lithium polysulfide conversion. Chem. Eng. J..

[103] Liu X, Wang S, Duan H, Deng Y, Chen G (2022). A thin and multifunctional CoS@g-C_3_N_4_/ketjen black interlayer deposited on polypropylene separator for boosting the performance of lithium–sulfur batteries. J. Colloid Interface Sci..

[104] Yao S, Wang Y, He Y, Majeed A, Liang Y (2020). Synergistic effect of titanium-oxide integrated with graphitic nitride hybrid for enhanced electrochemical performance in lithium–sulfur batteries. Int. J. Energy Res..

[105] Kim S, Shirvani-Arani S, Choi S, Cho M, Lee Y (2020). Strongly anchoring polysulfides by hierarchical Fe_3_O_4_/C_3_N_4_ nanostructures for advanced lithium–sulfur batteries. Nano-Micro Lett..

[106] Wang Y, Yang L, Chen Y, Li Q, Chen C (2020). Novel bifunctional separator with a self-assembled FeOOH/coated g-C_3_N_4_/KB bilayer in lithium–sulfur batteries. ACS Appl. Mater. Interfaces.

[107] Liang J, Yin L, Tang X, Yang H, Yan W (2016). Kinetically enhanced electrochemical redox of polysulfides on polymeric carbon nitrides for improved lithium–sulfur batteries. ACS Appl. Mater. Interfaces.

[108] Bian Z, Yuan T, Xu Y, Pang Y, Yao H (2019). Boosting Li–S battery by rational design of freestanding cathode with enriched anchoring and catalytic N-sites carbonaceous host. Carbon.

[109] Hong X, Liu Y, Fu J, Wang X, Zhang T (2020). A wheat flour derived hierarchical porous carbon/graphitic carbon nitride composite for high-performance lithium–sulfur batteries. Carbon.

[110] Wutthiprom J, Phattharasupakun N, Khuntilo J, Maihom T, Limtrakul J (2017). Collaborative design of Li–S batteries using 3D N-doped graphene aerogel as a sulfur host and graphitic carbon nitride paper as an interlayer. Sustain. Energy Fuels.

[111] Wutthiprom J, Phattharasupakun N, Sawangphruk M (2018). Designing an interlayer of reduced graphene oxide aerogel and nitrogen-rich graphitic carbon nitride by a layer-by-layer coating for high-performance lithium sulfur batteries. Carbon.

[112] Shu C, Fang L, Yang M, Zhong L, Chen X (2021). Cutting COF-like C_4_N into colloidal quantum dots toward optical encryption and bidirectional sulfur chemistry via functional group and edge effects. Angew. Chem. Int. Ed..

[113] Wu J, Wang LW (2018). 2D framework C_2_N as a potential cathode for lithium–sulfur batteries: an ab initio density functional study. J. Mater. Chem. A.

[114] Zheng Y, Li H, Yuan H, Fan H, Li W (2018). Understanding the anchoring effect of graphene, BN, C_2_N and C_3_N_4_ monolayers for lithium−polysulfides in Li−S batteries. Appl. Surf. Sci..

[115] Wang D, Li H, Zhang L, Sun Z, Han D (2018). 2D nitrogen-containing carbon material C_5_N as potential host material for lithium polysulfides: a first-principles study. Adv. Theory Simul..

[116] Dong Y, Xu B, Hu H, Yang J, Li F (2021). C_9_N_4_ and C_2_N_6_S_3_ monolayers as promising anchoring materials for lithium–sulfur batteries: weakening the shuttle effect via optimizing lithium bonds. Phys. Chem. Chem. Phys..

